# Effectiveness of intracuff alkalinized lidocaine associated with intravenous dexamethasone in reducing laryngotracheal morbidity in children undergoing general anesthesia for tonsillectomy: a randomized controlled trial

**DOI:** 10.1016/j.bjane.2024.844548

**Published:** 2024-08-03

**Authors:** Morenna Ramos e Oliveira, Norma S.P. Modolo, Paulo Nascimento, Rodrigo M. Lima, Devin Stirling, Glenio B. Mizubuti, Leopoldo Muniz da Silva, Lais H. Navarro

**Affiliations:** a Universidade Estadual Paulista (UNESP), Faculdade de Medicina de Botucatu, Departamento de Anestesiologia, São Paulo, SP, Brazil; b University of Manitoba, Department of Anesthesiology, Perioperative and Pain Medicine, Winnipeg, MB, Canada; c Queen's University, Department of Anesthesiology and Perioperative Medicine, Kingston, ON, Canada; d Hospital São Luiz, Departamento de Anestesiologia, Equipe de Anestesia do CMA, São Paulo, SP, Brazil

**Keywords:** Endotracheal intubation, Sore throat, Tonsillectomy, Pediatrics, Lidocaine

## Abstract

**Background:**

Postoperative sore throat is one of the main postoperative complaints in patients undergoing tonsillectomy. As the primary outcome, we aimed to determine whether endotracheal tube cuffs filled with alkalinized lidocaine are associated with a lower incidence of postoperative sore throat and anesthesia emergence phenomena in children undergoing tonsillectomy or adenotonsillectomy. We also assessed the potential additional benefits of IV dexamethasone in reducing postoperative laryngotracheal morbidity.

**Methods:**

This is a clinical prospective, randomized, controlled trial. Patients were randomly allocated to one of four groups, as follows: air **–** endotracheal tube cuff filled with air; air/dex – endotracheal tube cuff filled with air and intravenous dexamethasone; lido – endotracheal tube cuff filled with alkalinized lidocaine; and lido/dex – endotracheal tube cuff filled with alkalinized lidocaine and intravenous dexamethasone. Perioperative hemodynamic parameters and the incidence of postoperative nausea and vomiting, coughing and hoarseness were recorded. Postoperative sore throat was assessed in the postanesthetic care unit and 24 hours post tracheal extubation.

**Results:**

In total, 154 children aged 4–12 years, ASA physical status I or II, undergoing general anesthesia for elective tonsillectomy and adenotonsillectomy, were assessed for postoperative sore throat in this study. The incidence of postoperative sore throat 24 hours after tracheal extubation was significantly lower in the lido/dex group compared to groups air and air/dex (p = 0.01). However, no additional reduction in these symptoms was observed from the intravenous administration of dexamethasone when comparing the lido and lido/dex groups. Similarly, there were no differences among groups regarding perioperative hemodynamic variables or postoperative nausea and vomiting, coughing, and hoarseness during the study period.

**Conclusion:**

Intracuff alkalinized lidocaine, associated with intravenous dexamethasone, might be effective in reducing sore throat 24 hours post-tonsillectomy or adenotonsillectomy in children when compared to the use of air as the cuff insufflation media.

## Introduction

One of the main concerns among anesthesiologists is to reduce perioperative morbidity, especially those attributed to anesthesia. Accordingly, the prevention of tracheal intubation-induced sore throat has been the subject of extensive research.

In the past, pediatric intubation was classically performed using uncuffed Endotracheal Tubes (ETT) because of the potential risk of tracheal mucosa injury associated with cuffed ETT. However, in the early 2000s, the safety of ETT with high-compliance and low-pressure cuffs was demonstrated,^1^ as long as the tracheal mucosal perfusion pressure is not exceeded.^2^ Accordingly, the American Heart Association (AHA) and the International Liaison Committee on Resuscitation (ILCOR) established in 2010 the use of cuffed ETT as a safe alternative in children.^3^ Similarly, recent European guidelines for pediatric airway management recommend cuffed ETT for children undergoing tonsillectomy.^4^

Lately, researchers have been trying to find ways to reduce ETT-induced sore throat incidence. Some studies have demonstrated benefits when the ETT cuff is filled with alkalinized lidocaine (rather than air) in reducing adult postoperative laryngotracheal morbidity.^5^^,^^6^ A 2015 meta-analysis showed that both alkalinized and non-alkalinized lidocaine, when used to fill the ETT cuff, reduce the incidence of Postoperative Sore Throat (POST) and post-intubation emergence phenomena.^7^ The post-intubation-related emergence phenomena is a cluster of airway complications associated with tracheal intubation or extubation after general anesthesia.^7^ During emergence from general anesthesia, patients may experience vigorous coughing, agitation or restlessness. Other laryngeal complications, such as hoarseness, dysphonia, or dysphagia, may also be noted during postoperative care.^7^ Finally, a recent study also showed a reduction in POST (as well as a more favourable hemodynamic response to tracheal extubation) in pediatric patients when ETT cuffs were filled with alkalinized lidocaine.^8^ None of these studies, however, included children undergoing tonsillectomy or adenotonsillectomy.

POST is one of the main complaints in patients undergoing throat surgeries. Thus, different strategies have been employed to mitigate postoperative pain, including ntravenous (IV) corticoid administration,^9^ local anesthetic infiltration of the surgical site, and various surgical techniques.

Accordingly, as the primary outcome of the present study, we aimed to determine whether ETT cuffs filled with alkalinized lidocaine are associated with a lower incidence of POST and anesthesia emergence phenomena in children undergoing tonsillectomy or adenotonsillectomy. We also assessed the potential additional benefits of IV dexamethasone in reducing postoperative laryngotracheal morbidity.

## Methods

Local research ethics approval (protocol number: CAAE 4443-2012) and written informed consent from the patient's parents/legal guardians were obtained. This manuscript adheres to the applicable CONSORT guidelines and was registered at www.ensaiosclinicos.gov.br (RBR-9x4s6y) by one of the investigators (PN Jr) in October 2016.

This prospective, randomized, controlled trial was conducted in a Brazilian teaching hospital from November 2016 to May 2018. Children aged 4–12 years of both sexes, classified as American Society of Anesthesiologists (ASA) physical status I or II, undergoing elective tonsillectomy or adenotonsillectomy under general anesthesia, were eligible for inclusion.

Children with oropharyngeal or neck malformations; patients who presented with stridor or dysphonia before surgery; patients who had previously required tracheal intubation or tracheostomy; those requiring a nasogastric tube or more than two attempts at tracheal intubation; those with signs of upper respiratory tract infection; those whose parents did not sign the informed consent; and patients requiring postoperative Intensive Care Unit (ICU) admission (e.g., children with severe obstructive sleep apnea and hypopnea syndrome) were not included in the study. Moreover, patients who presented with severe intraoperative bleeding, need for reoperation, accidental extubation during surgical procedure, and change in the surgical plan were excluded from the final analysis.

An independent researcher allocated eligible patients to one of four groups: air ‒ ETT cuff filled with air and IV administration of saline 0.1 mL.kg^−1^ at anesthesia induction; air/dex ‒ ETT cuff filled with air and IV administration of dexamethasone 0.2 mg.kg^−1^ at anesthesia induction. Dexamethasone was diluted in order to achieve a volume of 0.1 mL.kg^−1^; lido ‒ ETT cuff filled with alkalinized lidocaine 1% and IV administration of saline 0.1 mL.kg^−1^ at anesthesia induction; lido/dex ‒ ETT cuff filled with alkalinized lidocaine 1% and IV administration of dexamethasone 0.2 mg.kg^−1^ at anesthesia induction. Dexamethasone was diluted in order to achieve a volume of 0.1 mL.kg^−1^.

The randomization list was generated using a computer sequence number, and the patient allocation ratio was 1:1:1:1. Written group allocation was sealed in individual opaque envelopes marked externally only with study identification numbers. Envelopes were opened upon the patient's admission to the Operating Room (OR). An anesthetic assistant not participating in the study prepared the drug solution after breaking the codes.

The alkalinized lidocaine solution was prepared by mixing 9.5 mL lidocaine 1% with 0.5 mL 8.4% sodium bicarbonate. The syringe containing dexamethasone or saline was prepared by a nurse, who was not directly involved in the study. The attending anesthesiologist, as well as the research team, were blinded to the type of IV solution administered to each patient. Polyvinyl chloride ETT with high-volume low-pressure cuffs (Well Lead Medical Instruments Co Ltd, Guangzhou, China) were used in all patients. The ETT size was chosen according to Motoyama's table^10^ and larger and smaller inner diameter ETT were also available.

Upon admission to the OR, standard monitors (electrocardiography, pulse oximeter, and non-invasive blood pressure) were applied, and anesthesia induction was carried out with 4%–5% sevoflurane in 100% oxygen via a facemask followed by peripheral intravenous line insertion. Fentanyl 3 mcg.kg^−1^ and propofol 3 mg.kg^−1^ were, then, administered, followed by rocuronium 0.5 mg.kg^−1^ to facilitate tracheal intubation. Upon full muscle relaxation (i.e., absence of response to Train-Of-Four [TOF]), direct laryngoscopy was performed and an appropriately-sized cuffed ETT with the cuff fully deflated and the tip lubricated with a water-soluble gel (Johnson & Johnson, New Brunswick, NJ, USA) was orally inserted into the trachea. The cuff was, then, filled with air or alkalinized lidocaine until the seal pressure was achieved during manual positive pressure ventilation with a pressure of 20 cm H_2_0. Mechanical ventilation was initiated with a tidal volume of 6–8 mL.kg^−1^. Anesthesia was maintained with sevoflurane (2 vol %) in an O_2_/N_2_O mixture of 40%‒60%. Minute ventilation was adjusted to maintain end-tidal CO_2_ (Et_CO2_) within 35 and 40 mmHg. The cuff pressure was continuously measured with a portable digital manometer (Mallinckrodt; Covidien, Dublin, Ireland) connected to the pilot-balloon valve in the air-filled cuff groups. In the lidocaine-filled cuff groups, the pressure was measured with a pressure transducer (Smith Medical ASD, Inc, Dublin, OH, USA) connected to a Dixtal DX 2023 monitor (Philips Healthcare, São Paulo, Brazil). Cuff pressure was maintained at < 20 cm H_2_0 throughout the case in all patients.

Hemodynamic (blood pressure and heart rate) and respiratory (pulse oximetry and Et_CO2_) parameters were recorded five minutes pre- and five minutes post-tracheal intubation, and every 15 minutes thereafter. Nitrous oxide was initiated upon tracheal intubation. ETT cuff pressure was first recorded upon tracheal intubation and then every 15 minutes thereafter.

Senior residents performed surgical procedures under the direct supervision of a board-certified otolaryngologist. The surgical technique (i.e., extracapsular cold dissection) was the same for all patients. Neither local anesthetics (topical or systemic) nor additional opioids were administered throughout the case.

Prior to the end of the surgical procedure, all patients receive IV ondansetron 0.1 mg.kg^−1^, metamizole 30 mg.kg^−1^ and tramadol hydrochloride 1 mg.kg^−1^. Antagonism of neuromuscular blockade (with sugammadex 2 mg.kg^−1^) was left at the discretion of the attending anesthesiologist, followed by careful oropharyngeal suction. Tracheal extubation was carried out once the following parameters were achieved: TOF ≥ 0.9; spontaneous ventilation with adequate tidal volumes; and response to verbal commands (eye-opening and movement of the limbs). At this point, the ETT cuff pressure was recorded and the cuff was completely deflated followed by tracheal extubation. Patients were transferred to the Postanesthetic Care Unit (PACU).

Hemodynamic variables were also recorded five minutes pre- and five minutes post-tracheal extubation. Postoperative laryngotracheal morbidity (namely coughing, hoarseness, and sore throat) was serially evaluated by the attending anesthesiologist upon tracheal extubation, and by blinded observers in the PACU and 24h post-tracheal extubation. Hoarseness was assessed subjectively by perceptual analysis of voice by the patient, the parent and the clinician. Nausea is the subjective unpleasant sensation of imminent vomiting. Accordingly, nausea was assessed by observation of gagging reflex or referral of nausea sensation by the child in the PACU and 24 hours after extubation. Vomiting was also assessed at the same time points. In the PACU, pain was self-informed by the patient based on a numeric scale ranging from 0 (absence of pain) to 10 (worst imaginable pain), or evaluated according to the faces pain scale,^11^ when the child was too young to fully comprehend the numeric scale. If the child presented with pain ≥ 5, IV morphine 25 mcg.kg^−1^ was administered as a rescue drug with a repeat similar dose after 10 min if necessary. Once patients achieved a score ≥ 9 on the modified Aldrete-Kroulik scale, they were transferred to the ward, where analgesia consisted of oral metamizole 30 mg.kg^−1^ every 6 hours and tramadol hydrochloride 1 mg.kg^−1^ every 8 hours. All surgeries were performed in the afternoon and all patients were discharged in the morning of the following day.

Our primary outcome was the incidence of POST. Secondary outcomes included perioperative hemodynamic variability, postoperative nausea/vomiting, coughing, and hoarseness. In the PACU, data collection was carried out by an investigator blinded to the group allocation. After 24 hours of tracheal extubation, data collection was carried out by another investigator through telephone contact with the parent/legal guardian, when 4 questions were asked to determine the presence/absence of sore throat: 1) Did the child objectively complain of pain?; 2) Was it necessary to give any analgesic medication to the child?; 3) After hospital discharge, has the child been able to ingest liquids and food?; 4) How is the general status of the child?. Of note, the presence/absence of sore throat was determined based on children's responses and subjective analysis by the parents/legal guardians.

### Statistical analysis

As our primary outcome was the incidence of sore throat post-tonsillectomy, the sample size was calculated on the basis of a minimum difference of 12% in the frequency of POST among groups with ETT cuffs filled with saline or lidocaine in adults.^6^ Thirty-five patients per group were required to achieve a significance level of 0.05 and a power of 80%.

ANOVA was performed for quantitative variables that were compared between groups, followed by a Tukey's test when significance was detected, in the case of symmetric distribution. Otherwise, the comparisons were made by fitting a gamma distribution followed by a Wald posthoc test. A p-value of < 0.05 was considered statistically significant. Pearson Chi-Squared test was employed for categorical variables, and partitioning Chi-Square when the value of p < 0.05. To be able to gauge the significance of the results in view of the multiple statistical comparisons, we included the corrected alpha level according to sequential Bonferroni correction. The significance level was lowered appropriately (Bonferroni method) to reduce the probability of a type I error. Residual analysis was calculated to identify those specific cells making the greatest contribution to the Chi-Square test result. Statistical analysis was performed using the computer software SAS for Windows (V. 9.4).

## Results

**Figure 1:**
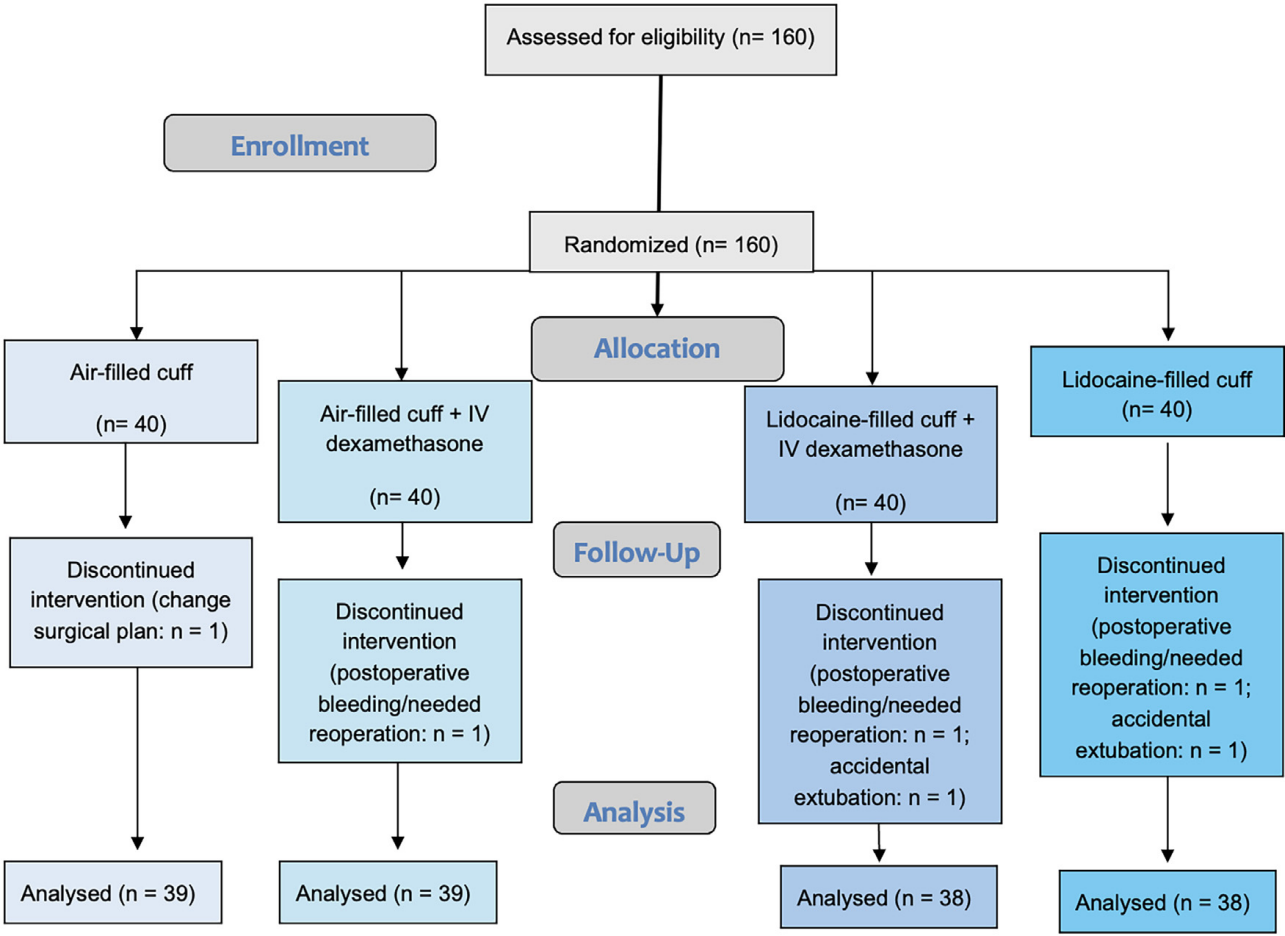
CONSORT flow of the study. CONSORT, Consolidated Standards Of Reporting Trials.

Figure 1 depicts the study flowchart. Of 160 eligible children, 154 were included in the final analysis. The four randomized groups were similar regarding morphometric and demographic data, as well as ASA physical status (Table 1). Likewise, surgical duration, time to extubation (from the end of surgery), and total time in PACU were similar among groups (Table 1).

**Table 1:** Preoperative demographic and morphometric patient characteristics, ASA physical status, surgical duration, time to extubation (from end of surgery), and time in PACU. Results expressed in Mean ± Standard Deviation (SD), or in frequency of occurrence (%).

	Groups				
Variables	Air	Air/Dex	Lido	Lido/Dex	p-value
Age (years)^a^	7.1 ± 2.6	6.8 ± 2.2	7.2 ± 2.4	6.3 ± 2.4	
Weight (kg)^b^	31.1 ± 14.7	30.7 ± 12.4	29.8 ± 12.7	28.7 ± 13.7	
Sex^c^					
Female	44%	59%	34%	37%	
Male	56%	41%	66%	63%	
ASA physical status^c^					
I	82%	87%	74%	82%	
II	18%	13%	26%	18%	
Surgery time (min)^a^	64.2 ±27.9	55.5 ± 20.8	57.7 ± 25.3	61.2 ± 23.1	0.42
Time to extubation (min)^a^	12.3 ± 0.7	15.9 ± 8.4	14.6 ± 6.2	15.1 ± 7.8	0.13
Time in PACU (min)^a^	49.5 ± 27.8	52.3 ± 27.3	46.7 ± 23.2	47.4± 19.3	0.71

a ANOVA followed by Tukey multiple comparison test.

b Adjust in gamma distribution followed by Wald multiple comparison test.

c Chi-Square test. G, Group; PACU, Post Anesthesia Care Unit.

The incidence of POST 24h after tracheal extubation was significantly lower in the lido/dex group, compared with groups air and air/dex (Table 2); (χ^2^ = 5.76; p = 0.010). The largest residual to the chi-square test was observed in the lido/dex group. No significant difference was observed when comparing groups air, air/dex and lido (χ^2^ = 2.99; p = 0.08). There was also no difference when comparing only groups lido and lido/dex (χ^2^ = 0.84; p = 0.35). No difference was observed among groups regarding nausea, vomiting and hoarseness during the study period.

**Table 2:** Laryngotracheal morbidity evaluated 24h post tracheal extubation. Results are expressed in frequency of occurrence (%).

	Groups				
Variables	Air	Air/Dex	Lido	Lido/Dex	p
**Sorethroat**^a^	74%	74%	58%	47%^b^	0.01
(Residual analysis)	−1.61	−1.61	0.84	2.40	
**Nausea**	5%	20%	16%	5%	0.08
**Vomiting**	3%	18%	13%	8%	0.13
**Hoarseness**	21%	13%	11%	16%	0.64

Values expressed as % (n).

a Proportion comparison test (Chi-Square) – Partitioning test: Incidence of sore throat: Lido/Dex group < Air, Air/Dex (χ^2^ = 5.76; p = 0.010).

b Lido group = Air and Air/Dex groups (χ^2^ = 2.99; p = 0.08); Lido group = Lido/Dex group (χ^2^ = 0.84; p = 0.35). A Bonferroni corrected p-value set statistical significance at p ≤ 0.017.

Hemodynamic variables recorded pre- and post-tracheal extubation are shown in Table 3. There were no differences among groups regarding hemodynamic variables at anesthesia emergence.

**Table 3:** Hemodynamic variables before and after tracheal extubation in the studied groups. Results expressed in Mean ± SD.

	Groups				
Variables	Air	Air/Dex	Lido	Lido/Dex	p-value
**Systolic blood pressure**					
Before extubation	104.5 ± 12.6	104.4 ± 12.7	103.8 ± 11.8	101.1 ± 10.8	0.56
After extubation	124.3 ± 18.2	120.9 ± 14.0	123.6 ± 14.2	122.6 ± 16.3	0.80
Variation	19.4 ± 14.9	16.1±10.8	19.8 ± 13.9	21.5 ± 15.4	0.38
**Diastolic blood pressure**					
Before extubation	56.9 ± 10.5	57.0 ± 10.5	56.1 ± 10.8	53.2 ± 10.9	0.37
After extubation	70.5 ± 13.1	65.2± 10.8	70.2 ± 13.2	66.7 ± 14.6	0.20
Variation	13.3 ± 11.8	8.0 ± 7.6	14.1 ± 14.7	13.5 ± 16.4	0.14
**Heart rate (beats.min)**					
Before extubation	98.2 ± 17.6	99.3 ± 18.1	97.2 ± 19.7	97.9 ± 19.3	0.97
After extubation	124.3 ± 18.2	123.2 ± 21.7	127.1 ± 20.6	124.5 ± 22.6	0.87
Variation	25.5 ± 19.3	23.3 ± 20.4	29.9 ± 24.8	26.6 ± 24.5	0.62

ANOVA followed by Tukey multiple comparison test.

In PACU, low incidence (and no difference among groups) of sore throat, nausea/vomiting, coughing and hoarseness were observed (Table 4).

**Table 4:** Laryngotracheal morbidity evaluated in PACU. Results are expressed in frequency of occurrence (%).

	Groups				
Variables	Air	Air/Dex	Lido	Lido/Dex	p-value
Sore throat	33%	26%	24%	26%	0.79
Nausea	5%	5%	5%	3%	0.93
Vomiting	5%	5%	5%	0%	0.56
Coughing	5%	10%	8%	5%	0.79
Hoarseness	2%	5%	3%	8%	0.64

Proportion comparison test (Chi-Square).

PACU, Post-Anesthesia Care Unit.

Mean ETT cuff pressures were kept below 20 cm H_2_0 throughout the case in all groups. The analyzed patients had no adverse effects or harms related to the ETT.

## Discussion

The present study demonstrated a significant reduction in the incidence of POST at 24h post-extubation among children who underwent tonsillectomy and received a combination of IV dexamethasone and cuff insufflation with alkalinized lidocaine. However, such interventions were not able to attenuate the hemodynamic changes during emergence from anesthesia, nor were they effective in reducing the incidence of other laryngotracheal morbidities, such as postoperative coughing and hoarseness.

Laryngotracheal injury has many risk factors, namely ETT cuff hyperinflation (leading to ischemic injury), traumatic/prolonged intubation, multiple attempts at intubation, and surgical manipulation. In our study, cuff pressure < 20 cm H_2_O was maintained, which is considered a safe threshold for ischemic airway injury in children.^1^

Lidocaine is a reasonable analgesic strategy to prevent POST as it blocks tracheal nociceptors.^11^ Previous studies in adults^5^^,^^6^ have demonstrated, by analysis of blood samples collected during surgical procedures, that alkalinized lidocaine was detectable in serum as early as 10 minutes after its instillation in the ETT cuff and the median concentration of serum lidocaine ranged from 1.12 to 1.52 mcg.mL^−1^, similar to the concentration achieved 120 minutes after intracuff lidocaine instillation (1.10 to 1.6 mcg.mL^−1^). Another study involving pediatric patients^8^ encountered a serum concentration of 0.08 mcg.mL^−1^ after 30 minutes following the application of 1% alkalinized lidocaine into the ETT cuff.

After diffusing through the ETT cuff membrane,^12^ lidocaine is able to block voltage-gated sodium channels of tracheal nociceptors, suppressing peripheral noxious transmission.^13^ This localized effect has been shown to improve tolerance to ETT and reduce the incidence of POST and hemodynamic response to tracheal extubation in adults^5^ and children.^8^ A Cochrane systematic review and meta-analysis confirmed the effectiveness of intracuff lidocaine for the prevention of POST in adults.^13^ In our study, however, the incidence of POST was reduced only at 24h after tracheal extubation, which cannot be explained by the topical/local effect of lidocaine, given its duration of action of < 2 hours in children.^14^ Rather, we speculate that systemic absorption of the intracuff lidocaine may have contributed to the better analgesia observed on the first postoperative day. Indeed, systemic lidocaine has analgesic and anti-inflammatory properties by inhibiting potassium and calcium channels and G-protein-coupled receptors^15^ which may last for weeks.^16^ Also, at serum concentrations of 1–20 mcg.mL^−1^ lidocaine reversibly suppresses the tonic action potential discharge of acutely injured nerves, contributing to pain relief.^16^ Notably, our group had previously measured serum lidocaine concentrations in adults^5^^,^^6^ and children^8^ and demonstrated that plasma absorption of effective concentrations of the local anesthetic (> 1 mcg.mL^−1^) occurs as quickly as 10 min following its application in the ETT cuff.^5^^,^^6^

A previous study in children undergoing adenotonsillectomy found a similar incidence of coughing and hemodynamic changes upon anesthesia emergence between IV versus intracuff lidocaine.^17^ The authors, however, did not alkalinize the lidocaine solution. In our study, sodium bicarbonate was added to the lidocaine solution, which increases the pH, making it more diffusible through the cuff membrane.^18^

Corticosteroids are well known for their antiemetic and anti-inflammatory properties. Dexamethasone, in particular, is an effective, safe, and inexpensive option to reduce postoperative emesis and pain in children undergoing tonsillectomy.^9^ We opted to administer dexamethasone at the induction of anesthesia, given its higher efficacy when compared to later administration.^19^ Nevertheless, in our study, we did not observe significant reduction in POST with dexamethasone. This may be due to the fact that the drug's anti-inflammatory effect may take several hours to appear. Some authors,^9^ for example, have demonstrated a significant reduction in POST after tonsillectomy not earlier than on the second postoperative day. Perhaps this same difference would have been detected by our group if we had also evaluated POST 48 hours post-surgery.

In the present study, the only group that benefited from a significant reduction in POST 24h post-surgery received a combination of IV dexamethasone and cuff instillation with alkalinized lidocaine. There was, however, no significant decrease in POST when these interventions were instituted in isolation. Hence, our study supports the concept of multimodal analgesia which has been strongly advocated for the pediatric population.^20^ The combination of different strategies allows for more effective analgesia with decreased opioid requirements, thereby minimizing the incidence/severity of side effects. The combination of dexamethasone and intracuff lidocaine, therefore, is a cheap and effective alternative with a potential synergistic effect with other analgesics as part of the multimodal management of post-tonsillectomy sore throat.

A 2018 RCT showed that dexamethasone associated or not with lidocaine was effective in reducing the incidence of POST in patients requiring prolonged tracheal intubation.^21^ Their patient population (adults undergoing all types of surgery) and route (intravenous) of lidocaine administration, however, differed from our study. Another study suggested that local anesthetic did not reduce the number of supplemental analgesics required post-tonsillectomy, but recommended the routine use of IV dexamethasone.^22^

Currently, IV dexamethasone is a well-established and recommended strategy to prevent POST in children undergoing tonsillectomy. However, the benefits of combining dexamethasone with lidocaine remain the subject of debate. Some authors advocate for such a combination on the basis of a synergistic effect resulting in more effective and prolonged benefits.^23^^,^^24^ Others, on the other hand, have shown no difference when these agents are administered either in combination or separately.^21^^,^^25^

To our knowledge, the present study is the first to evaluate the combination of IV dexamethasone and intracuff alkalinized lidocaine in comparison to such interventions in isolation. As the benefits were greater when used in combination, we speculate that IV dexamethasone potentialized the systemic effect of lidocaine. Nonetheless, further studies are needed to evaluate the synergistic effect of these two interventions in preventing POST in different surgical settings. Moreover, other studies are necessary to evaluate the incidence of POST 48 h post tracheal extubation.

Finally, we observed a low incidence of sore throat in the PACU. This may be due to the residual effect of anesthetic/analgesic agents administered intraoperatively. There was no difference among groups regarding hemodynamic changes upon anesthesia emergence. This corroborates our hypothesis that the effect of lidocaine was more systemic than local.

Our study has some limitations. First, it was not possible to determine whether the reported throat pain was supraglottic (surgical site) or infraglottic (ETT cuff site). However, the clinical benefit on the first postoperative day was clearly noted, either way. Secondly, as the study was conducted in pediatric patients, some of the information regarding pain was subjectively provided by parents. Without objective pain scales, parents may underestimate their child's pain. Further studies are needed to evaluate the effectiveness of an adapted pain scale for parent observation in children with acute pain. Moreover, we did not measure lidocaine serum concentrations. However, as plasma diffusion of intracuff lidocaine has been previously demonstrated, we opted to spare our pediatric patients from the stress associated with serial blood sampling.

In conclusion, intracuff alkalinized lidocaine, associated with IV dexamethasone, may reduce POST 24h post-tonsillectomy/adenotonsillectomy in children. Considering the lack of similar studies in other settings, the present study warrants further investigation of possible ways to mitigate laryngotracheal morbidity in pediatric patients.

## Conflicts of interest

The authors declare no conflicts of interest.
